# Retrospective Continuous-Time Blood Glucose Estimation in Free Living Conditions with a Non-Invasive Multisensor Device

**DOI:** 10.3390/s19173677

**Published:** 2019-08-24

**Authors:** Giada Acciaroli, Mattia Zanon, Andrea Facchinetti, Andreas Caduff, Giovanni Sparacino

**Affiliations:** 1 Department of Information Engineering, University of Padova, 35131 Padova, Italy; 2 Biovotion AG, 8008 Zurich, Switzerland

**Keywords:** diabetes, continuous glucose monitoring, non-invasive, multisensor

## Abstract

Even if still at an early stage of development, non-invasive continuous glucose monitoring (NI-CGM) sensors represent a promising technology for optimizing diabetes therapy. Recent studies showed that the Multisensor provides useful information about glucose dynamics with a mean absolute relative difference (MARD) of 35.4% in a fully prospective setting. Here we propose a method that, exploiting the same Multisensor measurements, but in a retrospective setting, achieves a much better accuracy. Data acquired by the Multisensor during a long-term study are retrospectively processed following a two-step procedure. First, the raw data are transformed to a blood glucose (BG) estimate by a multiple linear regression model. Then, an enhancing module is applied in cascade to the regression model to improve the accuracy of the glucose estimation by retrofitting available BG references through a time-varying linear model. MARD between the retrospectively reconstructed BG time-series and reference values is 20%. Here, 94% of values fall in zone A or B of the Clarke Error Grid. The proposed algorithm achieved a level of accuracy that could make this device a potential complementary tool for diabetes management and also for guiding prediabetic or nondiabetic users through life-style changes.

## 1. Introduction

Non-invasive continuous glucose monitoring (NI-CGM) has been widely investigated in the last years [1,2,3,4,5] because it would obviously represent an appealing technology to monitor glucose changes with no discomfort related to the use of subcutaneous needles [6,7,8] or implantable devices [6]. Although in terms of accuracy and reliability, the performance of NI-CGM is still far from that of commercial minimally-invasive CGM sensors, some encouraging results have been demonstrated in strictly controlled conditions during in-clinic sessions [5,9,10,11]. However, the use of NI-CGM technology in uncontrolled conditions met in daily life has shown several critical aspects, mostly related to the influence of external perturbations, e.g., environmental factors and non-glucose related physiological confounders that influence the sensor measurements [10,11,12,13,14,15].

**Figure 1:**
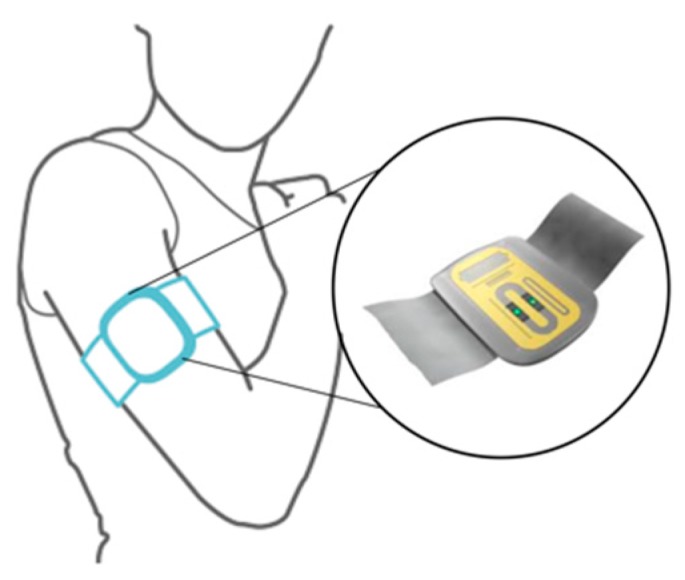
The Multisensor system worn on the upper arm and schematic illustration of its substrate.

One interesting approach systematically evolved by Caduff et al. proposed to mitigate the effect of such perturbing factors is the multisensor concept, i.e., the embedment of glucose and non-glucose-related sensors in the same device [11,15]. The basic idea behind the multisensor approach is to measure perturbing effects together with the glucose-related effects to allow for proper compensation via suitable modelling [14,15]. In particular, Solianis Monitoring AG (Zurich, Switzerland) acquired by Biovotion AG (Zurich, Switzerland), proposed and developed a multisensor solution, hereafter referred to as Multisensor and depicted in Figure 1, embedding a combination of dielectric and optical sensors [11]. The Multisensor is worn on the upper arm and implements black-box statistical models to combine the measured dielectric and optical signals into a glucose estimation, providing useful information about glucose dynamics in controlled and semi controlled conditions, as extensively documented in past recent literature [11,16,17,18].

The most recent clinical study conducted with the Multisensor in free-living conditions showed a point accuracy of the estimated glucose versus a reference glucose measurement, computed as mean absolute relative difference (MARD), of 35.4% [18]. These results were obtained considering a fully prospective estimate of glucose profiles by a global model, with only an initial calibration when the device is worn on the upper arm. The less accurate results, when compared with the current state-of-the-art minimally invasive CGM devices [6] and the accuracy obtained with the Multisensor in controlled conditions [19], is related to the sub optimality of the models and the algorithmic routines that may not yet properly compensate for all the extrinsic (environmental) and intrinsic (physiological) perturbations typical of the uncontrolled conditions mentioned above. These limitations currently prevent the actual use of the Multisensor in real-time diabetes management. Here, we aim at assessing the performance of the Multisensor system in a less challenging scenario, i.e., in retrospectively estimating the blood glucose (BG) concentration given the raw sensor measurements and a few self-monitoring BG (SMBG) samples that serve as a reference.

## 2. Materials and Methods

### 2.1. Database

The database was acquired during a long-term study conducted with the Multisensor device [20] involving 20 patients with type 1 diabetes (13 male, 7 female, Caucasian origin, age 38 ± 13 years, bod mass index, BMI, 24.1 ± 3.0 kg m^−2^, duration of diabetes 17.0 ± 13.0 years, HbA1c 7.5 ± 0.9%). Table 1 reports the subjects’ demography and characteristics in finer detail. The study was performed in accordance with Good Clinical Practice and the Declaration of Helsinki.

**Figure 2:**
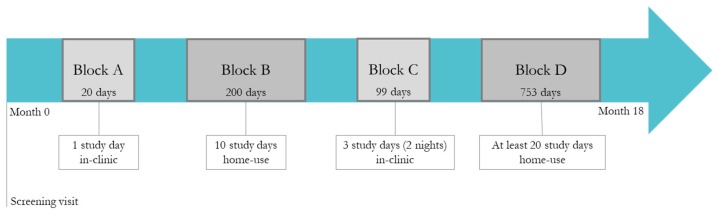
Schematic illustration of the study protocol. The study started with a screening visit at month 0 and ended after 18 months. Block A and C correspond to in-clinic sessions, while block B and D correspond to home-use of the device.

Figure 2 shows a graphical representation of the study procedure. After the initial screening visit (month 0), subjects entered block A of the study procedure, which consisted of one day of in-clinic sessions per subject. Following block A, subjects entered block B of the study design, which consisted of 10 days of home-use. This period of home-use was followed by another in-clinic period of 3 days and 2 nights per subject (block C). Then, the study ended with block D, a second period of home-use of at least 20 days for each subject. In total, the study lasted for 18 months and globally the following study days (or runs) were acquired: 20 days in block A, 200 days in block B, 99 days in block C, and 753 days in block D, for a total of 1072 runs (data acquired during the two in-clinic nights were not used in our analysis). The median length of all runs in the study was between 10 and 11 h. The minimum, maximum, and median durations of the home-use study blocks across subjects was 11, 79, and 31 days for block B and 29, 183, and 119 for block D.

The in-clinic study days, i.e., the runs belonging to blocks A and C, include the following glucose data: blood samples taken routinely every 10 to 20 min via a venous catheter for BG reference using a HemoCue Glucose 201 + (HemoCue, Sweden); regular SMBG samples taken via finger pricking about every 60 min using an Ascensia Contour (Bayer, Switzerland) glucose monitor. The home-use days, i.e., the runs belonging to blocks B and D, include only the SMBG measurements, which were taken in free-living conditions. In total, during the 4 study blocks, 3431 HemoCue and 13,338 SMBG measurements were collected. Moreover, for each run in blocks A, B, C, and D, the raw data collected with the Multisensor were available for processing. The Multisensor device, which has been extensively described in previous studies (see, e.g., [21] and references quoted in) and is shown in Figure 1, embeds dielectric spectroscopy and optical modules, as well as temperature, humidity, and sweat sensors, plus a 3-axes inertial sensor. All sensor signals were measured and recorded every 20 s. The capabilities of the Multisensor system have been expanded in a separate development in the last few years with new algorithmic routines allowing the monitoring of vital signs such as heart rate, pulse oximetry, and heart rate variability within an upper arm worn subsystem of the Multisensor, focusing around optical sensing and now commercially termed Everion^®^ [22,23].

### 2.2. Method

**Figure 3:**
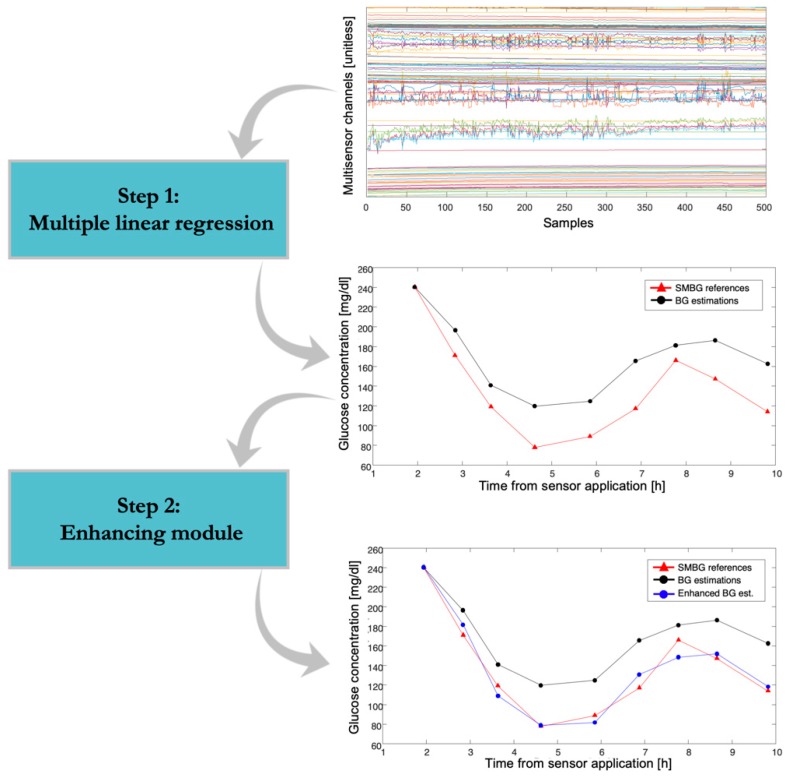
Schematic representation of the two-step procedure used to estimate the blood glucose (BG) time-series from the Multisensor measurements (real data from one representative subject). From top to bottom: raw Multisensor measurements, converted by the multiple linear regression (step 1) into a BG estimate (black curve, vs. reference measurements, red curve), further converted by the enhancing module (step 2) to enhanced BG estimate (blue curve, vs. BG estimate, black curve, vs. BG reference, red curve).

To retrospectively reconstruct the BG time-series from the raw Multisensor data and relative reference BG samples, we implemented a two-step procedure, illustrated in Figure 3. In step 1, the raw Multisensor data were transformed to a BG estimate through a multiple linear regression model. In step 2, an enhancing module was applied to improve the accuracy of the BG estimation obtained at step 1 by retrofitting available SMBG samples through a time-varying linear model. The two steps are described in detail in the following subsections.

#### 2.2.1. Step 1: Multiple Linear Regression

The multiple linear regression step implements the same model proposed in recent literature to estimate the BG from the Multisensor data [16,17,18]:

(1) y=Xβ+e,

where y is the n × 1 vector collecting the BG reference samples, X is the n × p matrix containing the Multisensor measurements, β the p × 1 vector of model coefficients, and e the n × 1 error vector, assumed to be independent and identically distributed.

The data collected in X depicted in Figure 3 (first panel), are characterized by high dimensionality and high correlation because most of the p channels of the Multisensor provide measurements of dielectric properties of the skin as a function of the frequency, thus exhibiting the correlated behavior typical of spectroscopy data. The problem of estimating β from Equation (1) is thus ill-conditioned, requiring a suitable regularization approach to control complexity and avoid overfitting. Here, we used the elastic net regularization, resulting in the following solution:

(2) $$\hat{\beta }=\underset{\beta }{\mathrm{argmin}}\left({\left\|y-X\beta \right\|}_{2}{}^{2}+\alpha {\left\|\beta \right\|}_{2}{}^{2}+\left(1-\alpha \right){\left\|\beta \right\|}_{1}\right),$$

where α is the hyper parameter balancing the linear and quadratic penalty terms (details on its estimation later in Section 2.3.1).

The model coefficients $\hat{\beta }$ were estimated from the training set, and then used to obtain the BG glucose prediction, $\hat{y}$, on an independent test set (training and test sets will be defined in Section 2.3.1):

(3) $$\hat{y}=X\hat{\beta }.$$

The estimated BG profile $\hat{y}$ was then scaled to match the first BG reference as in [16,17,18] (see second panel of Figure 3, dots and black curve, vs. SMBG references, triangle and red curve, from a representative subject). Note that, as a preprocessing procedure, the first 75 min of each Multisensor channel were removed from each run, since an adaptation process due to the Multisensor/skin contact dominates this time interval. Also, the Multisensor measurements in X were standardized to have unitary standard deviation (in the test set, the standard deviation estimated from the training set was used).

#### 2.2.2. Step 2: Enhancing Module

The enhancing module was applied in cascade to the multiple linear regression to obtain a more accurate glucose estimation, by exploiting the available SMBG references. In particular, given the BG estimation of step 1, $\hat{y}$, let z be the vector containing the samples in $\hat{y}$ corresponding to the time instants at which the SMBG references were collected. Then, the following model was used to relate the BG estimation given by the linear regression model to the reference BG:

(4) z=u+b+cΔt+w,

where u is the vector of SMBG reference values, w is the measurement noise, b and c are model parameters, and ∆t is the vector containing the times from sor application (in min) at which each SMBG was acquired. The model parameters b and c are estimated by least squaresens:

(5) $$\hat{b},\hat{c}=\underset{b,c}{\mathrm{argmin}}{\left\|z-u-b-c\Delta t\right\|}_{2}{}^{2}.$$

Then, the enhanced BG estimation, ${\hat{y}}_{\mathrm{en}}$, is obtained as follows:

(6) $${\hat{y}}_{\mathrm{en}}=z-\hat{b}-\hat{c}\Delta t.$$

The enhanced BG profile ${\hat{y}}_{\mathrm{en}}$ is shown in the third panel of Figure 3, blue curve, vs. the output of step 1 ($\hat{y}$, dots and black curve) and the SMBG references (triangle and red curve), for a representative subject. The enhancing module and, in particular, the use of the time-varying model of Equation (4) serves to adjust the point BG estimate by considering possible time-varying factors, e.g., environmental or non-glucose related phenomena that can influence the estimation. An example of this phenomenon is observable in Figure 3, second panel, where the BG estimation before the enhancing module shows a time-varying drift with respect to the reference BG. The application of the enhancing module, and, in particular, of the time-varying model of Equation (4), allows the compensation of such drift, as visible in the enhanced BG estimation profile reported in the bottom panel of Figure 3.

### 2.3. Implementation

#### 2.3.1. Dataset Subdivision into Training and Test Sets

To assess the method and fairly evaluate the performance, the entire dataset has been subdivided into training and test sets. The available data from blocks A, B, and C were used as a training set to estimate the regression model parameters $\hat{\beta }$ of Equation (2). In particular, we used all BG references acquired either with the EmoCue instrumentation or with the standard SMBG finger prick device for the estimation (i.e., in Equation (1), vector y contains all available BG references in the 20 subjects and matrix X contains the Multisensor measurements at corresponding times). In addition, the training data were also used to estimate the regularization hyperparameter α in Equation (2) by minimizing the standard error as calculated during a 5-fold cross-validation internal to the training set (i.e., the training runs were divided into 5 folds; for each fold, the standard error was computed by applying the regression model estimated in the remaining 4 folds, for various values of α). The available data from block D (which, we remind, were data acquired during free-living conditions) were used as a test set to assess the method. Firstly, the linear regression step was applied to each run as in Equation (3). Then, the enhancing module was used as in Equation (6). The parameters $\hat{b}$ and $\hat{c}$ of the enhancing module were estimated, for each run, by fitting the vector u of available SMBG references. In particular, besides using all available SMBG references, we also tested different scenarios in which a different number of SMBG samples were considered (from 10 to 4 SMBG references per run), in order to assess the robustness of the method against the number of available reference points. To create the scenarios with fewer than the total number of available SMBG, we defined a uniform time grid with the desired frequency of SMBG per run (from 10 to 4 references per day) and selected the references closer to the defined sampling times.

#### 2.3.2. Comparison with the Current State-Of-The-Art Method

To assess the performance of our two-step method, we compared the accuracy of the reconstructed BG time-series to that obtained with the method discussed in [16,17,18,19] (hereafter referred to as the baseline method). It is based on a multiple linear regression model (like that used in step 1 of our two-step method) plus a one-point calibration to adjust the offset of the estimated BG profile by adding a constant value. In formal terms, with all variables as defined previously and k calibration constant, the baseline method is represented by:

(7) $$\hat{y}=X\hat{\beta }+k.$$

The value of the constant k was determined in a one-point calibration procedure by matching the first BG reference acquired after sensor application.

The baseline method as described in [16,17,18,19] is intended for real-time use. The comparison of our retrospective approach with this real-time approach would not be fair, since we would use more information than only one BG reference. Thus, to allow a fair comparison, we fed the baseline method with the same amount of information used by our method, i.e., in estimating k in Equation (7) we used the mean of all available BG references instead of using only the first BG collected. Note that, for the baseline method, the estimation of the regression model parameters $\hat{\beta }$ was conducted as in step 1 of our two-step method, with the same training–test subdivision of the dataset.

#### 2.3.3. Performance Metrics and Statistical Analysis

The performance of our method and of the baseline method was assessed in terms of accuracy by comparing the retrospectively reconstructed BG time-series under test and the SMBG references acquired at the same time instants, using standard metrics. In particular, we performed two different evaluations. When all SMBG references were used to retrospectively reconstruct the BG time-series, then the same SMBG points were also used for accuracy assessment (indeed, there are no other references available to compute accuracy). When instead only a subset of the available SMBG references were used in the estimation, accuracy was computed in the remaining subset.

For each run, we computed the mean absolute difference (MAD) and the MARD [24]. We then computed the percentage of data matching the SMBG standard International Organization for Standardization (ISO) 15197:2013 [25], i.e., the percentage of data falling within either 15 mg/dL from the reference measurement if the reference was lower than 100 mg/dL or within 15% of reference if reference was above 100 mg/dL (15/15%). We also considered two other ranges, 20/20% and 30/30%. Moreover, accuracy was assessed by computing for each run the percentage of data falling in zone A and zone B of the Clark Error Grid (CEG) [26]. Finally, the population mean (standard deviation) was reported for normally distributed metrics and population median [interquartile range] was reported for non-normally distributed metrics. Normality was assessed by the Lilliefors test.

The statistical significance of the differences in performance metrics obtained with our method and with the baseline method was determined by a Wilcoxon signed-rank test for non-normally distributed data and by a paired t-test for normally distributed data. In particular, we tested the null hypothesis that the median/mean (in case of non-normally/normally distributed data) difference between the paired values of the two groups was zero, with a significance level of 0.05.

## 3. Results

**Figure 4:**
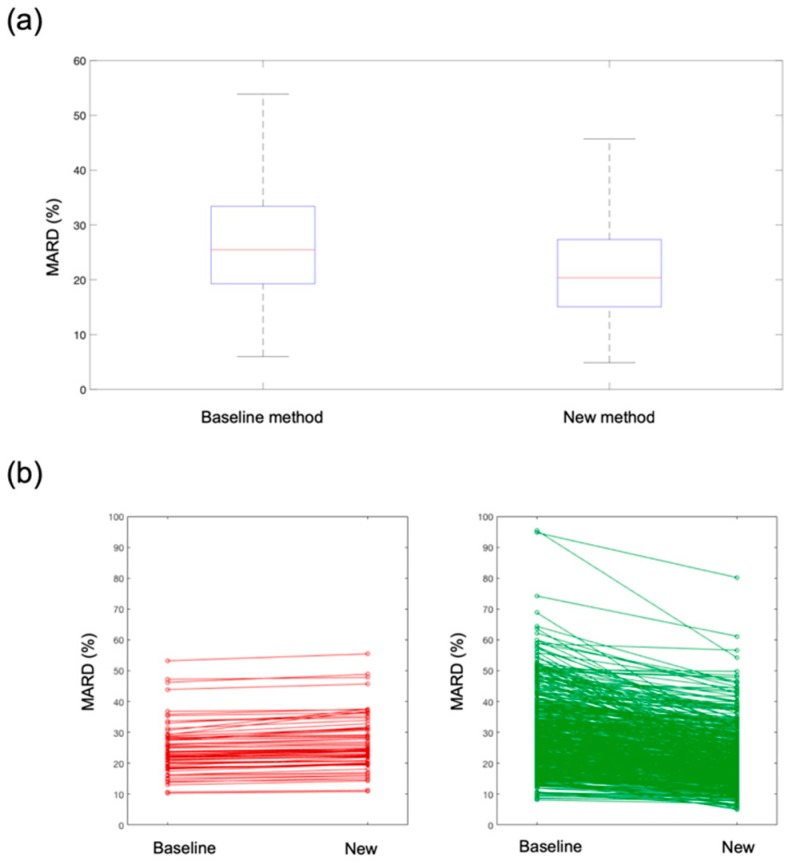
(**a**) Boxplot of MARD distributions in the population when using the baseline method (left boxplot) and the new method (right boxplot); (**b**) change in MARD for each single run when using the baseline and the new method. Left plot: runs with a MARD deterioration (~5% of the runs). Right plot: runs with a MARD improvement respect to the baseline method (~95% of the runs).

Table 2 shows the performance metrics of the new method compared to the baseline method when all available SMBG references were used to retrospectively reconstruct the BG time-series. The average number of SMBG samples used was 10 per run. The new method shows superior performance compared to the baseline method for all the considered metrics. Also, the improvement brought by the new method was statistically significant for all performance metrics (*p*-values < 10^−3^, not shown). In particular, focusing on metric MARD, which is one of the most popular metrics used in recent literature, a significant improvement from 25% to 20% (*p*-value < 10^−10^) was observed. A boxplot of the MARD distribution in the population is reported in Figure 4a, where the left boxplot refers to the baseline method and the right boxplot to the new method, showing a significant MARD reduction. This global performance improvement was also observable at a single-run level.

In Figure 4b, we depicted how the MARD changes in each single run under test when passing from the baseline method to the new method. In particular, in the left plot we reported (in red) the runs in which the MARD does not improve with the new method, while in the right plot we reported (in green) the runs that show a MARD improvement with the new method. As clearly observable from the figure, there are only a few runs (about 5% of all test runs) in which the MARD shows a very slight deterioration (runs in left panel, in red). On the contrary, the majority of the test runs (runs in right panel, in green) shows a significant improvement with the new method.

**Figure 5:**
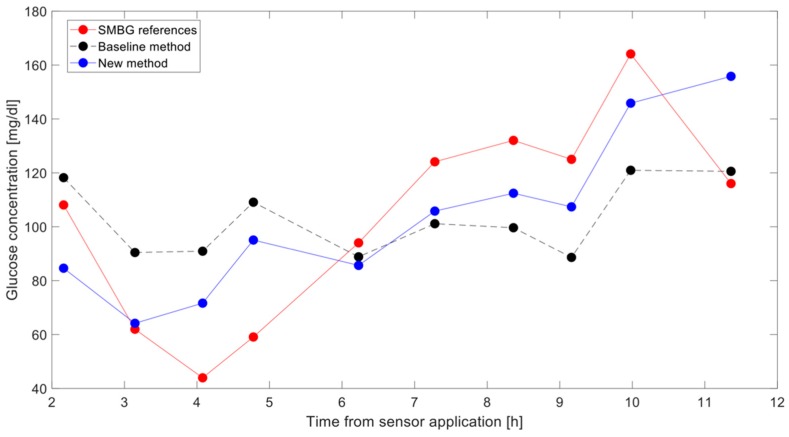
Reconstructed BG time-series with the baseline method (dashed black line) and with the new method (continuous blue line) versus self-monitoring BG (SMBG) references (red line) in a representative run and subject.

In Figure 5, we reported the estimated BG time-series versus the reference SMBG measurements in a representative subject and run. It is observable how the BG estimation obtained with the new method (blue line) is much closer to the reference SMBG samples (red line) than the BG estimation obtained with the baseline method (black line). Indeed, MARD values of this specific run and subjects were 35% and 25% with the baseline and new method, respectively.

After having demonstrated the superiority of the present method compared to the state-of-the-art baseline method, we also assessed the robustness of the new method against the number of SMBG references used to retrospectively reconstruct the BG time-series. In particular, we implemented the enhancing module (step 2 of the procedure described in Section 3) by varying the number of SMBG references from 10 to 4 per run.

Results of this assessment are reported in Table 3. Although, as expected, there was a slight deterioration of performance when reducing the number of SMBG references used for the estimation, the method appears relatively robust against the number of references available.

Indeed, the performance of the new method was always superior to that of the baseline method, independently from the number of SMBG references used (see the comparison reported for each performance metric in Table 3). Also, with the new method, even in the case in which only four SMBG samples were used, 90% of the estimations were in zone A or B of the CEG.

## 4. Discussion

Wearable non-invasive technologies for blood glucose monitoring, although widely investigated, still show important accuracy issues that hinder their use in a prospective real-time scenario. One of the major issues that contribute to sensor inaccuracy is the influence of external perturbations, e.g., environmental factors and non-glucose-related physiological confounders, on sensor measurements. The Multisensor system aims at mitigating the effect of such perturbing factors by embedding both glucose and non-glucose-related sensors in the same device and compensating for those via a multiple linear regression model [16,17,18,19]. We refer to this approach as the baseline method for BG estimation with the Multisensor.

A recent study showed that the Multisensor accuracy in estimating BG in free-living conditions and in a fully prospective scenario was 35.4% MARD [18]. The same prospective scenario, when applied in strictly controlled conditions, showed instead 21.1% MARD [19], confirming that the environmental and physiological perturbations typical of the uncontrolled conditions significantly impact sensor accuracy and thus need more sophisticated compensation.

Acknowledging the current existing accuracy limitations in utilizing the Multisensor for prospective (i.e., online) BG estimation, we analyzed here the less challenging, but still interesting, scenario of retrospective (i.e., offline) BG estimation. A retrospective approach allows the use of a few BG references to match the Multisensor estimation and compensate for those sources of bias (caused by physiological and/or environmental factors) that we may currently not be able to capture/describe with the Multisensor measurements/models.

In the retrospective approach presented in this paper, we have developed an enhancing module to be applied in cascade to the Multisensor regression model. The enhancing module consists of a time-varying linear model fit to a few SMBG references per day. To compare our results with the baseline method, we considered a retrospective implementation of the baseline method where we used exactly the same amount of information to feed the models, i.e., the same Multisensor input and the same SMBG references. The new method here proposed shows improved accuracy compared to the retrospective implementation of the baseline method, independently from the number of SMBG references used. When an average of 10 SMBG references per day was used as input data, MARD was reduced from 25% (baseline method) to 20% (new method). Also, the new method maintains superior performance compared to the baseline method when the number of SMBG inputs was reduced from 10 to 4 per day (30% MARD vs. 25% MARD).

The increased accuracy brought by the enhancing module attached in cascade to the regression model came at the cost of no more prospective applicability. However, we believe that a retrospective BG estimation from a non-invasive wearable device can still be useful for various applications. First of all, to estimate quality of glycemic control, magnitude of glycemic variability, daily patterns of hypo- and hyperglycemia, and time of day with the highest risks of hypo- or hyperglycemia events. Secondly, the retrospectively estimated BG profile and relative identified patterns can be subsequently used to make long-term changes in diet, medications, insulin, and physical activity [27]. Such applications are of interest not only for people with diabetes, but also to guide prediabetic or nondiabetic people through life-style changes towards healthier habits [28].

## 5. Conclusions

Given their potential advantages in terms of economic cost and user acceptability, NI-CGM devices can represent an interesting tool to investigate. In recent studies, encouraging results of NI-CGM have been shown in controlled situations, but their use in free-living conditions still represents a challenge, mostly because of inaccuracy problems related to the influence of intrinsic and extrinsic perturbing factors that can have adverse effects on the sensor measurements.

In the present study we assessed the accuracy of a multisensor device for NI-CGM in the less challenging, but still useful application, of retrospective, off-line, BG estimation. We proposed a two-step procedure in which, at step 1, the Multisensor measurements were combined into a BG estimate by multiple linear regression, and at step 2 an enhancing module, which represents the major novelty of this work, was used to improve the BG estimation. The method proved effective in retrospectively reconstructing the BG time-series, showing improved performance compared to a current state-of-the-art method used as a baseline for comparison. In particular, MARD was reduced from 25% of the baseline method to 20% of the new method.

Although the point accuracy was still significantly worse than that of minimally invasive CGM sensors, the BG estimations obtained by retrospectively processing the Multisensor data could be combined and used as additional and complementary information to the vital signs already provided by Everion^®^. It provides medical grade heart rate, blood oxygenation and perfusion, respiration rate, and further derived parameters [22,23,29], with the aim of facilitating effective glucose control in patients with diabetes but also in prediabetic or nondiabetic users that can benefit from this additional information for life-style changes, such as diet, weight loss programs, or physical exercise.

## Figures and Tables

**Table 1:** Subjects’ description.

Subject	Age [years]	BMI [kg/m^2^]	Diabetes Onset [year]	HbA1c [%]	Pump Carrier	Gender
AB04	52	23.3	1971	7.2	Yes	female
AB06	38	27.4	1998	7.6	No	male
AB07	28	22.6	1995	8.9	No	male
AB09	28	24.7	1997	7.5	Yes	female
AB10	54	20.0	1972	6.4	Yes	female
AB12	21	24.4	2000	6.9	Yes	male
AB13	54	25.2	1970	8.3	Yes	male
AB16	29	22.1	1982	7.3	No	male
AB18	22	23.5	2005	7.3	No	female
AB19	18	26.0	1993	9.5	Yes	male
AB23	23	24.8	2004	8.7	No	female
AB24	35	27.1	1985	7.4	No	male
AB25	37	20.6	1991	7.3	Yes	female
AB28	24	24.3	2008	5.5	Yes	male
AB30	58	21.5	2005	7.4	No	male
AB31	45	24.1	2002	7.7	Yes	female
AB32	54	24.2	1976	6.5	Yes	male
AB34	36	24.8	2000	8.0	No	male
AB35	32	26.3	1999	6.4	No	male
AB37	46	25.2	2007	7.5	Yes	male

**Table 2:** Performance metrics as median [interquartile range] or mean (standard deviation). MAD: mean absolute difference, MARD: mean absolute relative difference, CEG: Clark Error Grid.

	Pairs (#)	MAD (mg/dl)	MARD (%)	15/15% (%)	20/20% (%)	30/30% (%)	CEG-A (%)	CEG-A + B (%)
Baseline method	10 [3]	29 [16]	25 [14]	40 [27]	54 [28]	75 [29]	54 [29]	92 [26]
New method	10 [3]	24 [13]	20 [12]	50 [28]	64 [28]	82 [19]	64 [28]	94 [25]

**Table 3:** Performance metrics as median [interquartile range] or mean (standard deviation).

Method	SMBG	#pairs {#runs}	MAD (mg/dl)	MARD (%)	15/15% (%)	20/20% (%)	30/30% (%)	CEG-A (%)	CEG-A + B (%)
New Baseline	4/run	5 {723}	29 [16] 35 [18]	25 [18] 30 [20]	43 [35] 35 [32]	56 [35] 46 [36]	75 [26] 64 [23]	50 [35] 41 [32]	90 [33] 83 [35]
New Baseline	5/run	5 {546}	27 [18] 34 [17]	24 [19] 29 [19]	45 [35] 36 (34)	54 [35] 45 [35]	75 [40] 65 [30]	50 [35] 40 (30)	83 [33] 75 [33]
New Baseline	6/run	5 {333}	28 [16] 34 [18]	24 [16] 28 (20)	43 [35] 35 [32]	56 [35] 45 (33)	75 [40] 65 [30]	50 [35] 42 [31]	83 [33] 75 [33]
New Baseline	7/run	5 {205}	28 [18] 34 [16]	25 [19] 29 [19]	40 [35] 34 [30]	50 [35] 42 [34]	75 [40] 64 (35)	50 [35] 41 [32]	83 [33] 73 (35)
New Baseline	8/run	5 {123}	28 [20] 35 (17)	25 [20] 28 (19)	50 [35] 39 (30)	57 [35] 46 [35]	75 [28] 64 [28]	50 [35] 40 [34]	87 [33] 77 [31]
New Baseline	9/run	5 {72}	26 [17] 34 [18]	27 (13) 30 [18]	50 [38] 40 [32]	54 [32] 44 (30)	76 [21] 64 [28]	50 [32] 42 (32)	75 [32] 67 [30]
New Baseline	10/run	5 {38}	29 (13) 35 (18)	22 [19] 28 (19)	50 [35] 40 [32]	67 [25] 55 [30]	80 [40] 68 (30)	63 [35] 53 [32]	95 (22) 85 [31]

## References

[1] Vaddiraju S., Burgess D.J., Tomazos I., Jain F.C., Papadimitrakopoulos F. (2010). Technologies for Continuous Glucose Monitoring: Current Problems and Future Promises. J. Diabetes Sci. Technol..

[2] Ben Mohammadi L., Klotzbuecher T., Sigloch S., Welzel K., Göddel M., Pieber T.R., Schaupp L. (2014). In vivo evaluation of a chip based near infrared sensor for continuous glucose monitoring. Biosens. Bioelectron..

[3] Chen C., Zhao X.-L., Li Z.-H., Zhu Z.-G., Qian S.-H., Flewitt A.J. (2017). Current and Emerging Technology for Continuous Glucose Monitoring. Sensors.

[4] Pandey R., Paidi S.K., Valdez T.A., Zhang C., Spegazzini N., Dasari R.R., Barman I. (2017). Noninvasive Monitoring of Blood Glucose with Raman Spectroscopy. Acc. Chem. Res..

[5] Arnold M.A., Small G.W. (2005). Noninvasive glucose sensing. Anal. Chem..

[6] Cappon G., Acciaroli G., Vettoretti M., Facchinetti A., Sparacino G. (2017). Wearable continuous glucose monitoring sensors: A revolution in diabetes treatment. Electronics.

[7] Lane J.E., Shivers J.P., Zisser H. (2013). Continuous glucose monitors. Curr. Opin. Endocrinol. Diabetes Obes..

[8] McGarraugh G. (2009). The Chemistry of Commercial Continuous Glucose Monitors. Diabetes Technol. Ther..

[9] Larin K.V., Eledrisi M.S., Motamedi M., Esenaliev R.O. (2002). Noninvasive Blood Glucose Monitoring with Optical Coherence Tomography: A pilot study in human subjects. Diabetes Care.

[10] Caduff A., Dewarrat F., Talary M., Stalder G., Heinemann L., Feldman Y. (2006). Non-invasive glucose monitoring in patients with diabetes: A novel system based on impedance spectroscopy. Biosens. Bioelectron..

[11] Caduff A., Mueller M., Megej A., Dewarrat F., Suri R.E., Klisic J., Donath M., Zakharov P., Schaub D., Stahel W.A. (2011). Characteristics of a multisensor system for non invasive glucose monitoring with external validation and prospective evaluation. Biosens. Bioelectron..

[12] Caduff A., Talary M.S., Zakharov P. (2010). Cutaneous Blood Perfusion as a Perturbing Factor for Noninvasive Glucose Monitoring. Diabetes Technol. Ther..

[13] Zakharov P., Dewarrat F., Caduff A., Talary M.S. (2010). The effect of blood content on the optical and dielectric skin properties. Physiol. Meas..

[14] Amaral C.F., Brischwein M., Wolf B. (2009). Multiparameter techniques for non-invasive measurement of blood glucose. Sens. Actuators B Chem..

[15] Harman-Boehm I., Gal A., Raykhman A.M., Naidis E., Mayzel Y. (2010). Noninvasive Glucose Monitoring: Increasing Accuracy by Combination of Multi-Technology and Multi-Sensors. J. Diabetes Sci. Technol..

[16] Zanon M., Sparacino G., Facchinetti A., Riz M., Talary M.S., Suri R.E., Caduff A., Cobelli C. (2012). Non-invasive continuous glucose monitoring: Improved accuracy of point and trend estimates of the Multisensor system. Med. Boil. Eng..

[17] Zanon M., Sparacino G., Facchinetti A., Talary M.S., Caduff A., Cobelli C. (2013). Regularised Model Identification Improves Accuracy of Multisensor Systems for Noninvasive Continuous Glucose Monitoring in Diabetes Management. J. Appl. Math..

[18] Zanon M., Muller M., Zakharov P., Talary M.S., Donath M., Stahel W.A., Caduff A. (2018). First experience with a wearable multisensor device in a noninvasive continuous glucose monitoring study at home, part II: The invesigators’ view. J. Diabetes Sci. Technol..

[19] Caduff A., Zanon M., Mueller M., Zakharov P., Feldman Y., De Feo O., Donath M., Stahel W.A., Talary M.S. (2015). The Effect of a Global, Subject, and Device-Specific Model on a Noninvasive Glucose Monitoring Multisensor System. J. Diabetes Sci. Technol..

[20] Caduff A., Zanon M., Zakharov P., Mueller M., Talary M., Krebs A., Stahel W.A., Donath M. (2018). First Experiences with a Wearable Multisensor in an Outpatient Glucose Monitoring Study, Part I: The Users’ View. J. Diabetes Sci. Technol..

[21] Dewarrat F., Falco L., Caduff A., Talary M.S., Feldman Y., Puzenko A. (2008). Measurement and simulation of conductive dielectric two-layer materials with a multiple electrodes sensor. IEEE Trans. Dielectr. Electr. Insul..

[22] Barrios L., Oldrati P., Santini S., Lutterotti A. Evaluating the accuracy of heart rate sensors based on photoplethysmography for in-the-wild analysis. Proceedings of the 13th EAI International Conference on Pervasive Computing Technologies for Healthcare, PervasiveHealth 2019.

[23] Barrios L., Santini S., Oldrati P., Lutterit A. Recognizing Digital Biomarkers for Fatigue Assessment in Patients with Multiple Sclerosis. Proceedings of the 12th EAI International Conference on Pervasive Computing Technologies for Healthcare.

[24] Wentholt I., Hart A., Hoekstra J., Devries J.H. (2008). How to Assess and Compare the Accuracy of Continuous Glucose Monitors?. Diabetes Technol. Ther..

[25] (2013). ISO 15197:2013: In Vitro Diagnostic Test Systems–Requirements for Blood-Glucose Monitoring Systems for Self-Testing in Managing Diabetes Mellitus.

[26] Clarke W.L. (2005). The Original Clarke Error Grid Analysis (EGA). Diabetes Technol. Ther..

[27] Roadbard D. (2016). Continuous glucose monitoring: A review of successes, challenges, and opportunities. Diabetes Technol. Ther..

[28] Vettoretti M., Cappon G., Acciaroli G., Facchinetti A., Sparacino G. (2018). Continuous Glucose Monitoring: Current Use in Diabetes Management and Possible Future Applications. J. Diabetes Sci. Technol..

[29] Beeler N., Roos L., Delves S.K., Veenstra B.J., Friedl K., Buller M.J., Wyss T. (2018). The Wearing Comfort and Acceptability of Ambulatory Physical Activity Monitoring Devices in Soldiers. IISE Trans. Occup. Ergon. Hum. Factors.

